# A retrospective study on the association between urine metanephrines and cardiometabolic risk in patients with nonfunctioning adrenal incidentaloma

**DOI:** 10.1038/s41598-022-19321-2

**Published:** 2022-09-01

**Authors:** Mirko Parasiliti-Caprino, Chiara Lopez, Martina Bollati, Fabio Bioletto, Chiara Sola, Maria Chiara Di Carlo, Federico Ponzetto, Iacopo Gesmundo, Fabio Settanni, Ezio Ghigo, Giulio Mengozzi, Mauro Maccario, Roberta Giordano

**Affiliations:** 1 Endocrinology, Diabetes and Metabolism, Department of Medical Sciences, City of Health and Science University Hospital, University of Turin, Corso Dogliotti 14, 10126 Turin, Italy; 2 Clinical Biochemistry Laboratory, City of Health and Science University Hospital, Turin, Italy; 3 Department of Biological and Clinical Sciences, University of Turin, Turin, Italy

**Keywords:** Endocrine system and metabolic diseases, Adrenal gland diseases, Metabolic syndrome

## Abstract

Several studies argued that cardiovascular evaluation of patients with nonfunctioning adrenal incidentaloma is of particular importance. Therefore, we aimed to evaluate the possibility of stratifying the cardiometabolic risk using metanephrine levels in this setting of patients. A retrospective cross-sectional study was designed, collecting data of metanephrine values in 828 patients with nonfunctioning adrenal incidentaloma, referred to our Division within the University of Turin between 2007 and 2021. The univariate analysis showed associations between urine metanephrines and cardiometabolic variables/parameters, particularly considering the noradrenaline metabolite. At the univariate regression, normetanephrine was associated with metabolic syndrome (OR = 1.13, p = 0.002), hypertensive cardiomyopathy (OR = 1.09, p = 0.026), microalbuminuria (OR = 1.14, p = 0.024), and eGFR < 60 mL/min/1.73 m^2^ (OR = 1.11, p = 0.013), while metanephrine was associated with microalbuminuria (OR = 1.50, p = 0.008). At multivariate regression, considering all major cardiovascular risk factors as possible confounders, normetanephrine retained a significant association with metabolic syndrome (OR = 1.10, p = 0.037). Moreover, metanephrine retained a significant association with the presence of microalbuminuria (OR = 1.66, p = 0.003). The present study showed a further role for metanephrines in the cardiovascular risk stratification of patients with nonfunctioning adrenal incidentaloma. Individuals with high levels of these indirect markers of sympathetic activity should be carefully monitored and may benefit from an aggressive treatment to reduce their additional cardiometabolic burden.

## Introduction

An adrenal incidentaloma is an adrenal mass ≥ 1 cm, detected on imaging not performed for suspected adrenal disease^1^. In this setting, the diagnostic work-up consists in the exclusion of a malignant neoplasm (either primary or a metastatic tumor) and in the assessment of the hormone secretion, which are both potential indications for surgical treatment. An accurate hormone evaluation of gluco- and mineralocorticoid function, in some cases of androgenic secretion and in most cases of the catecholamine (CA) production is thus performed. Dopamine, noradrenaline and adrenaline derive from tyrosine metabolism^2^ and are adaptive/maladaptive stress hormones. The metanephrines, the urine and plasma metabolites of CA through the activity of Catechol-O-MethylTransferase (COMT), are nowadays the preferred markers for diagnosis and follow-up of pheochromocytoma and paraganglioma (PPGL)^3–5^. Metanephrines derive from non-neuronal sources (extra-neuronal and adrenomedullary pathways), because sympathetic nerves contain monoamine oxidase (MAO), but not COMT^2^. It has been suggested that metanephrines can be considered indirect markers of the whole sympathetic system activity^6^; moreover, a recent research of our group showed that, in patients without the evidence of PPGL, the elevation of metanephrines is associated with cardiometabolic complications, and that metanephrines could thus be useful in the stratification of cardiovascular (CV) risk^7^, highlighting the importance of a strict follow-up and an aggressive treatment of cardiometabolic risk factors in patients with a mild elevation of these metabolites.

In last years, the scientific community showed growing interest in the definition of the CV risk in patients with nonfunctioning adrenal incidentaloma (NFAI), even if a cause-effect relationship is not readily evident. In this setting, a non-detectable small amount of glucocorticoid excess would not completely justify the CV risk and the impairment of cardiometabolic profile seems to occur over a long time, leading to difficulties in studying this issue in a prospective manner.

Therefore, we aimed to determine if higher levels of urine metanephrines can be associated with CV and metabolic risk in patients with NFAI. In order to account for the physiologic variability of the sympathetic activity, we collected a large sample size, and the most commonly used risk scores were adopted as the assessment tool for CV risk.

## Materials and methods

### Design and study population

This retrospective cross-sectional study followed the STROBE statement for reporting observational studies^8^ and analysed all consecutive patients with adrenal incidentaloma, referred to the Division of Endocrinology, Diabetes and Metabolism of the City of Health and Science University Hospital of Turin, between September 2007 and September 2021. Patients < 18 years old, with chronic heart failure, arrhythmias, secondary hypertension (primary aldosteronism, pheochromocytoma/paraganglioma, autonomous cortisol secretion, overt hypercortisolism, obstructive sleep apnoea, renal artery stenosis, primary hyperparathyroidism and other causes), renal insufficiency with estimated glomerular filtration rate (eGFR) < 30 mL/min, psychiatric illness, liver cirrhosis, chronic diseases with major organ involvement, excessive alcohol ingestion, current assumption of sympathomimetics, cocaine and drugs commonly affecting the measurement of metanephrines (Acetaminophen, Labetalol, Sotalol, tricyclic antidepressants, Buspirone, Phenoxybenzamine, MAO inhibitors, Sulphasalazine and Levodopa) were excluded from the study.

Regarding the hormone evaluation of incidentaloma, patients with cortisol over 1.8 μg/dL after 1 mg dexamethasone suppression test (DST) and with aldosterone-to-renin ratio (ARR) > 300 [(pg/mL)/(ng/mL/h)] or with ARR > 200 [(pg/mL)/(ng/mL/h)] with signs and symptoms of mineralocorticoid hypertension were excluded. The basal aldosterone cut-off for considering the suspicion of primary aldosterone was 150 pg/mL.

An accurate endocrinological evaluation (repeating the dosages of metanephrine, adding Chromogranin A and Neuron Specific Enolase, and even performing functional imaging techniques, where appropriate) and follow-up (every 6–12 months, if necessary) were performed for patients with metanephrine values over the upper cut-off limits. Patients diagnosed with PPGL were excluded, checking data from the registry of the Piedmont Oncological Network to avoid the risk of including small PPGL with mild/non-secretive phenotype.

Data were collected within prospective registries and analysed retrospectively. The study was performed in accordance with the guidelines in the Declaration of Helsinki and approved by the Ethics Committee of City of Health and Science University Hospital of Turin (no. 0035241). Written informed consent was obtained from all enrolled patients (ClinicalTrials.gov no. 04495231).

### Clinical and biochemical investigations

Personal and clinical data, familial history of arterial hypertension, diabetes mellitus (DM) and early cardiovascular event, office blood pressure (BP) values^9^, serum levels of glucose, lipid profile, sodium, potassium, creatinine, values of 24-h urinary metanephrines, aldosterone, plasma renin activity (PRA) and cortisol after 1 mg dexamethasone suppression test (DST) were collected. Measurement of CA metabolites was performed on single or double 24-h urine collection. In the case of two or more dosages, we considered the value of the first collection. Cardiovascular risk was estimated using risk calculator (Framingham risk score^10^, Progetto CUORE^11^ and SCORE^12^).

### Evaluation of metabolic syndrome and organ damage

Metabolic syndrome was defined according to the ATP III criteria^13^. Organ damage was defined according to the 2018 ESC/ESH guidelines. eGFR was estimated using CKD-EPI formula and microalbuminuria was defined between 30 and 300 mg/24 h or by an albumin to creatinine ratio of 30–300 mg/g. Left ventricular mass index, assessed by echocardiography, was calculated with the formula: 0.8 * 1.04 * [(interventricular septum + left ventricular internal diameter + inferolateral wall thickness)^3^ − left ventricular internal diameter^3^] + 0.6 gr; in normal weight subjects, this value was indexed for body surface area, and left ventricular hypertrophy was defined by a left ventricular mass index > 115 g/m^2^ for men and > 95 g/m^2^ for women; in overweight and obese subjects, it was indexed for height, and left ventricular hypertrophy was defined by a left ventricular mass index > 50 g/m^2.7^ for men and > 47 g/m^2.7^ for women^9^.

### Analytical methods

As previously described^14^, 24-h urinary metanephrines (normal ranges: normetanephrine 105–354 μg/day, metanephrine 74–298 μg/day) were measured by chromatographic determination on isocratic high-performance liquid chromatography (HPLC) system with electrochemical detector fixed with a potential of 740 mV (Chromsystems Instruments & Chemicals GmbH, Gräfelfing, Germany). Briefly, 1 mL of 24 h urine samples were mixed with 100 µl Internal Standard and poured in sealable hydrolysis tubes that were incubated for 30 min at 90°–100° in a water bath. Then a neutralization buffer was added to samples, and the entire neutralized urines were applied to sample clean up columns for a solid-phase extraction, that were successively mixed, centrifuged, and washed. Amount (20–50 μl) of eluates were injected into the HPLC system, and the retention times of normetanephrine and metanephrine and Internal Standard were respectively 5.5, 7.0 and 8.4 min. Furthermore, limits of quantification, intra-assay and inter-assay coefficients of variation were respectively: 5 mg/L, 1.4% and 2.7% for normetanephrine and 11 mg/L, 1.8% and 2.8% for metanephrine.

### Statistical analysis

Statistical analysis of this study was similar to another research published by our group^7^, but the studied population was completely different from the previous paper, as it clear from the above sections. Baseline characteristics of all patients with NFAI included in the analysis were summarized using mean and standard deviation (SD), after the analysis of each variable/parameter through the quantile–quantile (Q-Q) plot, considering the large sample size. Binary and categorical data were reported using percent values. In the descriptive statistics, the sample was divided according to the tertiles of normetanephrine and metanephrine. The division into tertiles was decided because it allowed to have an adequate number of patients in each tertile, thus reinforcing the power of the statistical analysis. Between groups differences in personal and clinical features were evaluated by one-way ANOVA for continuous variables and chi-square test or Fisher’s exact test for categorical variables, as appropriate.

Associations between the levels of normetanephrine/metanephrine and the presence of cardiometabolic/renal complications (hypertensive cardiomyopathy, metabolic syndrome, eGFR < 60 ml/min/1.73 m^2^ and albuminuria) were assessed through univariate and multivariate logistic regressions, considering all the risk factors known to be possibly related with adverse cardiovascular or renal outcomes as possible confounders.

In this analysis, metanephrines were considered as continuous variables, and the coefficients of the models (with correspondent odds ratios) were computed by considering 100 μg/day as their unitary increase. A p-value < 0.05 was considered statistically significant. Statistical analysis was performed using R 3.5.3 (R Core Team, R Foundation for Statistical Computing, Vienna, Austria, 2019).

## Results

**Figure 1:**
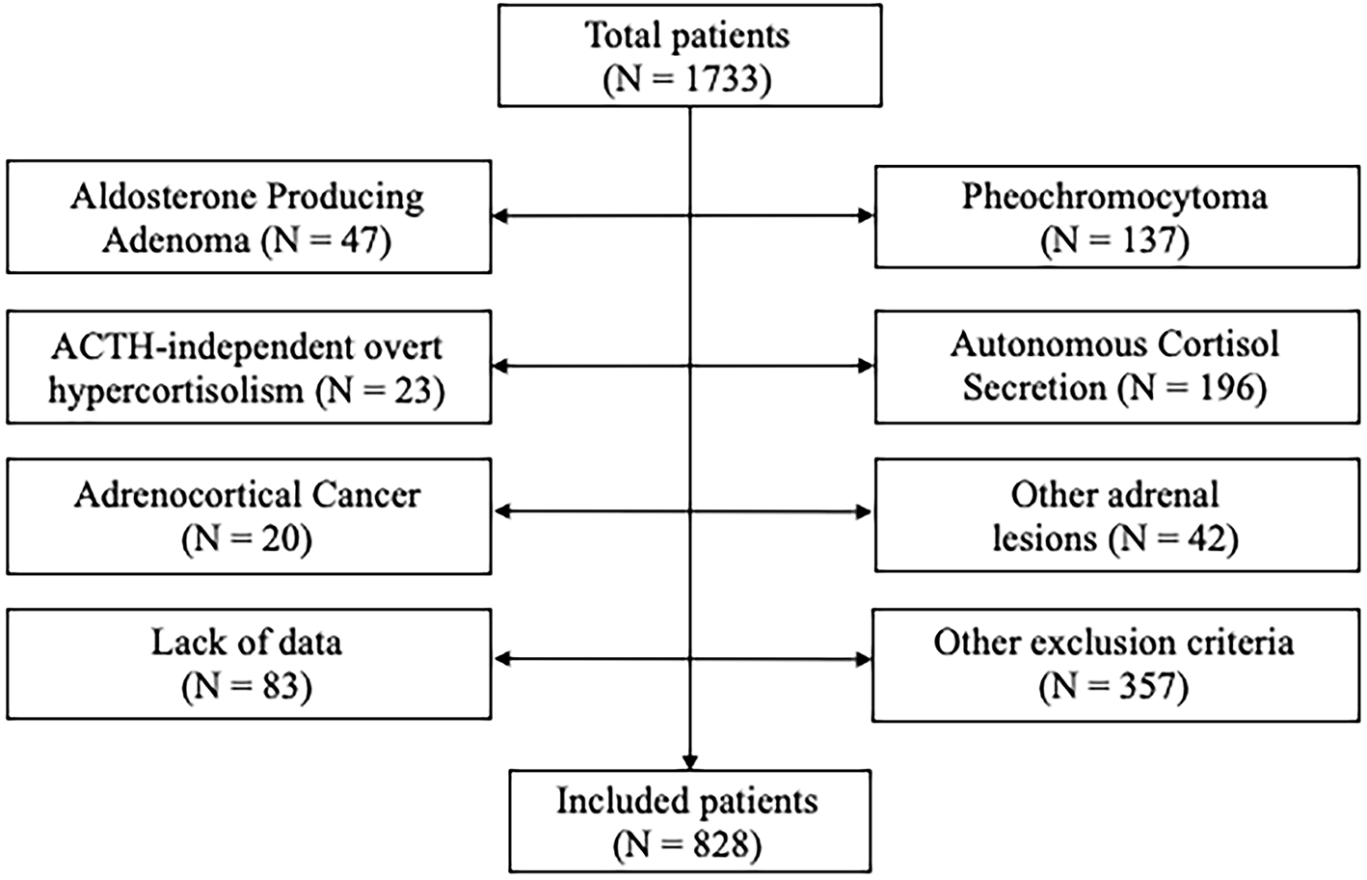
Study flow-chart. PPGL, pheochromocytoma or paraganglioma.

After the application of the inclusion/exclusion criteria, as shown in Fig. 1, 828 patients with NFAI were enrolled in the study (324 males—39.1% and 505 females—60.9%). The sample was divided into tertiles of normetanephrine (I tertile: 20.0–248.0 μg/day; II tertile: 249.0–388.0 μg/day; III tertile: 390.0–2158.0 μg/day) and metanephrine (I tertile: 10.0–69.0 μg/day; II tertile: 69.2–119.0 μg/day; III tertile: 119.6–510.0 μg/day).

When comparing patient characteristics among tertiles, patients with higher levels of urine normetanephrine proved to be older (p = 0.002), had higher proportion of male sex (p < 0.001), smoking habit (p = 0.037), obesity (p = 0.014), history of hypertensive crisis (p = 0.007), metabolic syndrome (p = 0.002), hypertensive cardiomyopathy (p = 0.027), higher values of BMI (p < 0.001), weight (p < 0.001), waist circumference (p = 0.003), office systolic BP (SBP, p = 0.019), office diastolic BP (DBP, p = 0.021), Framingham risk score (p < 0.001), and lower proportion of familial history of arterial hypertension (p = 0.014), compared to patients with normetanephrine into the I-II tertiles.

Regarding the drug therapy, patients with high levels of normetanephrine were taking higher number of antihypertensive drugs (p = 0.003) and had higher proportion of treatment with beta-blockers (p = 0.028), α-blockers (p = 0.005), and angiotensin II receptor blockers (ARB, p = 0.024), if compared to patients with normetanephrine into the I-II tertiles.

A similar analysis showed also that individuals with higher values of urine metanephrine had higher proportion of male sex (p < 0.001), smoking habit (p = 0.004), hypertensive crisis (p = 0.003), and treatment with MRA or amiloride (p < 0.001), but lower proportion of obesity (p = 0.036), and therapy with thiazide (-like) diuretics (p = 0.002), compared to patients with metanephrines into the I-II tertiles. No differences were found in the remaining variables/parameters (Tables 1, 2, 3 and S1).

**Table 1:** Clinical variables/parameters according to tertiles of normetanephrine and metanephrine levels.

Variables/parameters	Overall (N = 828)	Normetanephrine				Metanephrine			
		I tertile	II tertile	III tertile	p-value	I tertile	II tertile	III tertile	p-value
Age (years)	59.5 ± 14.7	56.9 ± 16.5	60.9 ± 14.5	60.8 ± 12.6	0.002*^†^	60.2 ± 14.9	59.9 ± 15.1	58.4 ± 14.2	0.306
Male sex	39.1%	30.8%	36.6%	50%	< 0.001^†‡^	29.5%	37.1%	50.9%	< 0.001*^†‡^
Smoking habit	44.2%	37.9%	45.8%	49.2%	0.037^†^	39.8%	39.8%	52.6%	0.004^†‡^
FH of CVD	13.3%	13.1%	12.1%	14.7%	0.726	11.6%	13.6%	14.5%	0.644
FH of AH	45.1%	52.8%	40.3%	41.9%	0.014*^†^	45.0%	44.2%	46.3%	0.908
FH of DM	26.4%	24.5%	27.9%	27.0%	0.695	25.0%	27.4%	27.0%	0.833
Arterial hypertension	86.3%	85.5%	84.2%	89.2%	0.216	85.9%	87.0%	85.9%	0.916
Hypertensive crisis	6.3%	2.6%	6.8%	10.0%	0.007*^†^	1.9%	7.5%	9.6%	0.003*^†^
Weight (kg)	74.9 ± 16.3	71.4 ± 16.2	73.6 ± 14.7	79.8 ± 16.7	< 0.001	75.3 ± 17.9	73.8 ± 14.5	75.6 ± 16.1	0.41
BMI (kg/m^2^)	27.5 ± 5.5	26.5 ± 5.5	27.5 ± 5.6	28.5 ± 5.3	< 0.001*^†‡^	28.2 ± 6.2	27.3 ± 5	26.9 ± 5.3	0.032^†^
Waist circumference (cm)	101.5 ± 16.6	97.9 ± 14.4	95.9 ± 12.4	106.9 ± 18.4	0.003^†‡^	101 ± 15.1	102.1 ± 18.7	101.2 ± 16.2	0.947
Obesity	34.3%	30.4%	30.1%	42.6%	0.014^†‡^	38.1%	37.4%	27.1%	0.036^†‡^
Office SBP (mmHg)	133.8 ± 17.4	132.7 ± 15.3	132.5 ± 16.5	136.3 ± 19.8	0.019^†‡^	152.5 ± 15.1	134.8 ± 16.8	134.2 ± 20	0.316
Office DBP (mmHg)	80.6 ± 10.9	80.6 ± 9.6	79.2 ± 10.2	81.8 ± 12.7	0.021^‡^	80.5 ± 10.6	80.2 ± 10.3	80.9 ± 11.9	0.817
DM	17.5%	15.4%	18.9%	18.3%	0.539	20.4%	16.7%	15.3%	0.295
Prediabetes/DM	23.3%	19.7%	23.2%	27.1%	0.064^†^	25.1%	23.0%	21.6%	0.536
Other tumors	32.1%	27.6%	32.9%	35.9%	0.125^†^	32.4%	33.9%	30.2%	0.668
Antihypertensive drugs (no)	1.25 ± 1.26	1.07 ± 1.23	1.28 ± 1.24	1.42 ± 1.3	0.003*^†^	1.29 ± 1.27	1.22 ± 1.25	1.25 ± 1.27	0.806

AH, arterial hypertension; BMI, body mass index; CVD, cardiovascular disease; DBP, diastolic blood pressure; DM, diabetes mellitus; FH, familial history; SBP, systolic blood pressure.

The table shows p-values of the statistics analyzing differences between the three tertiles.

*Significant difference between I tertile and II tertile.

^†^Significant difference between I tertile and III tertile.

^‡^Significant difference between II tertile and III tertile.

**Table 2:** Biochemical, hormone and tumor-specific variables/parameters according to tertiles of normetanephrine and metanephrine levels.

Variables/parameters	Overall (N = 828)	Normetanephrine				Metanephrine			
		I tertile	II tertile	III tertile	p-value	I tertile	II tertile	III tertile	p-value
Glucose (mg/dL)	98.7 ± 31	98.7 ± 39.2	95.5 ± 22.8	102.3 ± 11.2	0.071‡	99.2 ± 37.3	99.3 ± 28.6	97.8 ± 25.7	0.859
Total cholesterol (mg/dL)	195.8 ± 52.7	194.1 ± 48	198.2 ± 54.6	195 ± 55.7	0.707	197.7 ± 50.8	197 ± 55.9	192.7 ± 51.4	0.584
Triglycerides (mg/dL)	125.0 ± 69.2	121.5 ± 86.6	124.4 ± 53.6	128.7 ± 61	0.576	125.5 ± 84.9	124.8 ± 58.3	124.9 ± 61.1	0.994
HDL cholesterol (mg/dL)	51.5 ± 16.1	52.8 ± 14.3	51.6 ± 15.8	49.8 ± 18.1	0.199	52.1 ± 13.7	51.9 ± 15.8	50.4 ± 18.5	0.522
LDL cholesterol (mg/dL)	120.2 ± 44.8	119.5 ± 41	122.2 ± 41.1	119.0 ± 52.4	0.768	123.1 ± 43.5	120.4 ± 53.1	117.1 ± 36.3	0.434
PRA (ng/mL/h)	3.60 ± 17.03	1.54 ± 2.06	1.46 ± 1.79	6.32 ± 25.56	0.202	1.57 ± 2.01	1.42 ± 1.56	8.63 ± 31.20	0.053‡
Aldosterone (pg/ml)	159 ± 104	174 ± 107	164 ± 119	145 ± 88	0.336	154 ± 100	171 ± 107	147 ± 104	0.433
Cortisol after 1 mg DST (μg/L)	10.0 ± 5.2	9.9 ± 5.3	10.0 ± 4.7	10.0 ± 5.5	0.990	10.1 ± 4.9	10.3 ± 5.1	9.3 ± 5.5	0.431
ECG HR (bpm)	74.4 ± 16.2	73.9 ± 15.4	73.9 ± 17.0	75.7 ± 16.3	0.569	73.9 ± 14.5	75.5 ± 18.1	74.1 ± 16.3	0.686
Tumor size (mm)	23.2 ± 13.4	23.3 ± 12.5	21.7 ± 11.3	24.3 ± 15.4	0.356	22.7 ± 11.0	23.6 ± 14.4	22.8 ± 14.3	0.868
Tumor side									
*Right*	43.3%	43.0%	37.0%	48.8%	0.335	44.1%	41.0%	45.7%	0.957
*Left*	47.2%	47.3%	55.0%	40.6%		47.1%	48.4%	45.7%	
*Bilateral*	9.5%	9.7%	8.0%	10.6%		8.8%	10.7%	8.7%	

DST, dexamethasone suppression test; eGFR, estimated glomerular filtration rate; HDL, high density lipoprotein; LDLc, low density lipoprotein calculated; PRA, plasma renin activity.

The table shows p-values of the statistics analyzing differences between the three tertiles.

^‡^Significant difference between II tertile and III tertile.

**Table 3:** Cardiovascular risk scores and cardiometabolic complications according to tertiles of normetanephrine and metanephrine levels.

Variables/parameters	Overall (N = 828)	Normetanephrine				Metanephrine			
		I tertile	II tertile	III tertile	p-value	I tertile	II tertile	III tertile	p-value
Framingham risk score (%)	9.3 ± 9.5	7.3 ± 7.7	9.6 ± 9.7	11.5 ± 10.7	< 0.001*^†‡^	8.4 ± 7.6	9.4 ± 11.3	10.2 ± 9.4	0.177^†^
SCORE (%)	3.8 ± 5.0	3.7 ± 5.9	3.7 ± 4.4	4.0 ± 4.4	0.853	4.2 ± 5.8	3.4 ± 4.4	3.7 ± 4.6	0.330
Progetto cuore (%)	12.3 ± 14.5	11.1 ± 15.1	12.1 ± 13.7	13.9 ± 14.5	0.182	12.8 ± 15.2	11.8 ± 14.9	12.2 ± 13.4	0.812
EF (%)	58.2 ± 9.6	58.6 ± 10.1	57.4 ± 9.7	58.5 ± 8.9	0.813	58.9 ± 9.2	57.4 ± 11.5	58.2 ± 8.8	0.801
Previous CVE	21.0%	19.6%	21.0%	22.5%	0.705	21.6%	21.3%	20.4%	0.934
Metabolic syndrome	46.1%	36.9%	48.8%	53.7%	0.002*^†^	46.9%	46.5%	45.0%	0.919
Items of metabolic syndrome									
*0 factors*	5.7%	6.9%	7.1%	2.6%	0.222	4.8%	6.1%	6.2%	0.987
*1 factor*	19.3%	23.5%	19.5%	14.3%		20.1%	17.7%	20.1%	
*2 factors*	28.9%	32.7%	24.8%	29.1%		28.2%	29.8%	28.7%	
*3 factors*	29.7%	24.4%	29.5%	36.0%		29.2%	28.3%	31.6%	
*4 factors*	12.7%	9.7%	15.7%	12.7%		13.4%	14.1%	10.5%	
*5 factors*	3.7%	2.8%	3.3%	5.3%		4.3%	4.0%	2.9%	
Microalbuminuria	5.8%	6.2%	6.0%	5.1%	0.901	5.4%	3.1%	8.6%	0.089^‡^
Creatinine (mg/dL)	0.9 ± 0.5	0.91 ± 0.61	0.87 ± 0.36	0.87 ± 0.31	0.617	0.89 ± 0.48	0.84 ± 0.25	0.92 ± 0.55	0.234
eGFR (CKD-EPI, mL/min/1.73 m^2^)	78.8 ± 23.4	81 ± 24.4	78.3 ± 22.5	77 ± 23.1	0.191	78.9 ± 24.0	80 ± 21.9	77.7 ± 24.1	0.624
eGFR < 60 mL/min	20.4%	17.9%	19.1%	24.7%	0.181	19.7%	18.1%	23.2%	0.414
Hypertensive cardiomyopathy	26.1%	19.4%	30.5%	28.9%	0.027*^†^	24.5%	22.9%	30.3%	0.221

Abbreviations: CKD-EPI, chronic kidney disease epidemiology collaboration; CVE, cardiovascular events; EF, ejection fraction; eGFR, estimated glomerular filtration rate.

The table shows p-values of the statistics analyzing differences between the three tertiles.

*Significant difference between I tertile and II tertile.

^†^Significant difference between I tertile and III tertile.

^‡^Significant difference between II tertile and III tertile.

### Univariate and multivariate logistic regressions

In the present study, urine metanephrines were analysed as possible independent variables associated to cardiometabolic and renal complications, considering all other common risk factors as potential confounders of these associations.

At univariate regression, normetanephrine proved to be associated to metabolic syndrome (OR = 1.13, 95% CI 1.05–1.22; p = 0.002), hypertensive cardiomyopathy (OR = 1.09, 95% CI 1.01–1.18; p = 0.026), microalbuminuria (OR = 1.14, 95% CI 1.02–1.25; p = 0.024), and with eGFR < 60 mL/min/1.73 m^2^ (OR = 1.11, 95% CI 1.02–1.19; p = 0.013) (Table 4).

**Table 4:** Univariate logistic regressions on the association of metanephrines with presence of metabolic syndrome and organ damage (ORs of normetanephrine and metanephrine are calculated for a unit of increase of 100 μg/day).

Dependent variables	Normetanephrine			Metanephrine		
	OR	95% CI	p-value	OR	95% CI	p-value
Metabolic syndrome	1.13	1.05–1.22	0.002	1.00	0.79–1.20	0.956
Hypertensive cardiomyopathy	1.09	1.01–1.18	0.026	1.09	0.85–1.33	0.455
Microalbuminuria	1.14	1.02–1.25	0.024	1.50	1.13–1.87	0.008
eGFR < 60 mL/min/1.73 m^2^	1.11	1.02–1.19	0.013	1.09	0.85–1.33	0.461

CI, confidence interval; eGFR, estimated glomerular filtration rate; OR, odds ratio.

At multivariate analysis, normetanephrine retained a statistically significant association with metabolic syndrome (OR = 1.10, 95% CI 1.01–1.19; p = 0.037), after correction for sex, age, smoking habit (OR = 1.63, 95% CI 1.12–2.39; p = 0.011), familial history of CVD, number of antihypertensive drugs (OR = 1.49, 95% CI 1.27–1.76; p < 0.001) and eGFR (Table 5).

**Table 5:** Logistic regression analysis on the association of metanephrines and covariates with presence of metabolic syndrome (ORs of normetanephrine and metanephrine are calculated for a unit of increase of 100 μg/day).

Covariates	Metabolic syndrome					
	OR	95% CI	p-value	OR	95% CI	p-value
Male sex	0.99	0.65–1.51	0.969	1.04	0.69–1.59	0.839
Age	1.00	0.99–1.02	0.653	1.00	0.99–1.02	0.582
Smoking habit	1.63	1.12–2.39	0.011	1.65	1.13–2.41	0.009
FH of CVD	1.23	0.68–2.21	0.487	1.22	0.68–2.18	0.502
No. of antihypertensive drugs	1.49	1.27–1.76	< 0.001	1.51	1.28–1.78	< 0.001
eGFR	1.00	0.99–1.01	0.517	1.00	0.99–1.01	0.502
*Normetanephrine*	1.10	1.01–1.19	0.037	–	–	–
*Metanephrine*	–	–	–	1.06	0.81–1.30	0.640

CI, confidence interval; CVD, cardiovascular disease; eGFR, estimated glomerular filtration rate; FH, family history; OR, odds ratio.

Urine normetanephrine was not significantly associated with presence of potentially hypertension-mediated organ damage, such as hypertensive cardiomyopathy (Table 6), microalbuminuria (Table 7) and impaired renal function (Table 8).

**Table 6:** Logistic regression analysis on the association of metanephrines and covariates with presence of hypertensive cardiomyopathy (ORs of normetanephrine and metanephrine are calculated for a unit of increase of 100 μg/day).

Covariates	Hypertensive cardiomyopathy					
	OR	95% CI	p-value	OR	95% CI	p-value
Male sex	1.95	1.23–3.10	0.004	2.00	1.25–3.21	0.004
Age	1.04	1.02–1.06	< 0.001	1.04	1.02–1.06	< 0.001
Smoking habit	1.21	0.78–1.91	0.397	1.22	0.78–1.9	0.394
FH of CVD	1.15	0.57–2.26	0.688	1.15	0.57–2.24	0.699
BMI	1.05	1.01–1.10	0.014	1.06	1.01–1.10	0.013
SBP	1.01	1.00–1.02	0.429	1.01	0.99–1.02	0.422
DBP	1.01	0.98–1.03	0.660	1.01	0.98–1.03	0.671
DM	1.68	0.95–2.93	0.071	1.68	0.95–2.92	0.073
No. of antihypertensive drugs	1.29	1.02–1.63	0.035	1.28	1.02–1.63	0.036
ACEi/ARB	1.17	0.66–2.05	0.593	1.18	0.67–2.07	0.562
*Normetanephrine*	1.04	0.94–1.13	0.438	–	–	–
*Metanephrine*	–	–	–	1.00	0.70–1.29	0.991

ACEi/ARB, angiotensin converting enzyme inhibitors or angiotensin II receptor blockers; BMI, body mass index; CI, confidence interval; CVD, cardiovascular disease; DBP, diastolic blood pressure; DM, diabetes mellitus; FH, family history; OR, odds ratio; SBP, systolic blood pressure.

**Table 7:** Logistic regression analysis on the association of covariates with presence of microalbuminuria (ORs of metanephrine are calculated for a unit of increase of 100 μg/day).

Covariates	Microalbuminuria					
	OR	95% CI	p-value	OR	95% CI	p-value
Male sex	1.41	0.58–3.43	0.758	1.10	0.96–2.77	0.757
Age	1.01	0.97–1.04	0.748	1.01	0.97–1.05	0.651
Smoking habit	0.77	0.32–1.78	0.537	0.71	0.30–1.69	0.445
FH of CVD	0.66	0.09–2.70	0.611	0.48	0.05–2.26	0.418
BMI	1.00	0.91–1.09	0.984	1.00	0.91–1.09	0.970
SBP	1.01	0.98–1.04	0.475	1.01	0.98–1.04	0.462
DBP	1.01	0.96–1.06	0.644	1.01	0.96–1.06	0.818
DM	6.00	2.35–15.24	< 0.001	6.82	2.63–18.03	< 0.001
eGFR	0.97	0.95–0.99	0.005	0.97	0.95–0.99	0.004
ACEi/ARB	3.30	0.64–1.80	0.054	3.65	1.09–12.92	0.039
*Normetanephrine*	1.10	0.96–1.24	0.147	–	–	–
*Metanephrine*	–	–	–	1.66	1.21–2.98	0.003

ACEi/ARB, angiotensin converting enzyme inhibitors or angiotensin II receptor blockers; BMI, body mass index; CI, confidence interval; CVD, cardiovascular disease; DBP, diastolic blood pressure; DM, diabetes mellitus; eGFR, estimated glomerular filtration rate; FH, family history; OR, odds ratio; SBP, systolic blood pressure.

**Table 8:** Logistic regression analysis on the association of metanephrines and covariates with presence of eGFR < 60 mL/min/1.73 m^2^ (ORs of normetanephrine and metanephrine are calculated for a unit of increase of 100 μg/day).

Covariates	eGFR < 60 mL/min/1.73 m^2^					
	OR	95% CI	p-value	OR	95% CI	p-value
Male sex	3.43	2.10–5.68	< 0.001	3.41	2.08–5.67	< 0.001
Age	1.07	1.04–1.09	< 0.001	1.07	1.05–1.09	< 0.001
Smoking habit	0.82	0.51–1.33	0.427	0.82	0.50–1.32	0.415
FH of CVD	0.85	0.38–1.79	0.683	0.83	0.37–1.76	0.648
BMI	1.01	0.96–1.06	0.706	1.01	0.96–1.06	0.623
SBP	1.01	0.99–1.02	0.427	1.01	0.99–1.02	0.448
DBP	1.00	0.97–1.03	0.915	1.00	0.97–1.03	0.922
DM	1.04	0.56–1.88	0.901	1.04	0.56–1.87	0.911
No. of antihypertensive drugs	1.14	0.92–1.40	0.234	1.14	0.92–1.40	0.223
*Normetanephrine*	1.05	0.95–1.15	0.282	–	–	–
*Metanephrine*	–	–	–	1.14	0.83–1.44	0.376

BMI, body mass index; CI, confidence interval; CVD, cardiovascular disease; DBP, diastolic blood pressure; DM, diabetes mellitus; eGFR, estimated glomerular filtration rate; FH, family history; OR, odds ratio; SBP, systolic blood pressure.

Regarding the adrenaline metabolite, at univariate logistic regression, metanephrine proved to be associated with microalbuminuria (OR = 1.50, 95% CI 1.13–1.87; p = 0.008); conversely, no significant associations with hypertensive cardiomyopathy, metabolic syndrome or eGFR < 60 mL/min/1.73 m^2^ were found (Table 4).

At multivariate analysis, metanephrine retained a statistically significant association with microalbuminuria (OR = 1.66, 95% CI 1.21–2.98; p = 0.003), considering sex, age, smoking habit, familial history of CVD, BMI, office SBP and DBP, DM (OR = 6.82, 95% CI 2.63–18.03; p < 0.001), eGFR (OR = 0.97, 95% CI 0.95–0.99; p = 0.004) and treatment with ACEi/ARB (OR = 3.65, 95% CI 1.09–12.92; p = 0.039), as covariates (Table 7).

Urine metanephrine was not associated with metabolic syndrome (Table 5), hypertensive cardiomyopathy (Table 6) or eGFR < 60 mL/min/1.73 m^2^ (Table 8).

To exclude interferences and potential sources of bias, the series was analyzed with the same statistics after excluding all patients treated with drugs affecting the sympathetic system. The results (data not shown) were not different from those previously reported.

## Discussion

In the present study, we found that high levels of 24-h urinary metanephrine levels are associated with CV risk and cardiometabolic complications in a large cohort of patients with NFAI. Particularly, high levels of normetanephrine proved to be independently associated with metabolic syndrome, while high metanephrine levels showed an independent association with microalbuminuria. Our study provided evidence on the possibility to stratify cardiovascular risk in patients with NFAI through metanephrine levels, because of their capability in the indirect assessment of the sympathetic activity and not only in the diagnosis of PPGL.

In patients with adrenal incidentaloma, the role of mild cortisol excess has been described as a possible cause of metabolic disorders, such as diabetes^15^, cardiovascular events^16^ and mortality^17^, even if the mechanisms that account for the link between cortisol and cardiovascular diseases need to be clarified. But in NFAI, the risk of developing autonomous cortisol secretion is debate and heterogenous in the literature^15,16,18^. Therefore, a low degree of cortisol excess would not completely justify the additional cardiovascular risk, that was recently described in this group of patients. Even if it seems difficult to prove an association between cardiometabolic complications and NFAI, several studies argued that the cardiovascular evaluation of patients with incidental adrenal findings is of particular importance. In fact, it has been reported that patients with NFAI had high prevalence of arterial hypertension, even resistant^19,20^, dyslipidemia^20^, insulin resistance^19–22^, type 2 diabetes mellitus^20,23^ and metabolic syndrome^21,24–26^. It is possible that increased risk for cardiometabolic diseases reported in NFAI patients is at least partially dependent on adipose tissue activity^27^, as showed by increased leptin and resistin levels^19^, although data on adiponectin regulation are contradictory^19,28^. Moreover, some authors demonstrated increased arterial stiffness^29–31^, epicardial fat thickness^32^, left ventricular mass^31–33^, carotid intima-media thickness^21,22,28,30,32,34,35^ and other markers of atherosclerosis^36^ in patients with NFAI, compared to healthy subjects.

It has been suggested that NFAI can manifest a slight excess of cortisol that cannot be detected by current diagnostic tests or intermittent hormonal secretion^29,37,38^. Some authors hypothesized also that these adrenal tumors can secrete a small amount of non-routinely or non-currently detectable steroids with a detrimental cardiovascular effect. In fact, patients with adrenal incidentalomas showed different steroid profiles, depending on functional activity and adrenal morphology, with potential implications for their cardiovascular status^39^. It has also to be noted that adrenal cortex and medulla are morphologically and functionally interwoven, through a strong endocrine and paracrine interaction^40^. Cellular interactions of chromaffin and cortical cells are critical in physiology and disease^41^. Glucocorticoids play a role in the development of norepinephrine-secreting cells into epinephrine-secreting cells by upregulating the expression of Phenylethanolamine N-MethylTransferase^42^. Oppositely, CA and/or neuropeptides secreted by the adrenal medulla have been suggested to stimulate the release of steroids and the cellular function of the adrenal cortex^40^. Moreover, it was shown that if the adrenal cortex is absent or impaired, there could be also consequences for the adjacent medullary tissue^43^. So, it is also possible that the elevation of metanephrines in patients with NFAI can be related to the presence of the adrenal tumor, revealing a direct link between NFAI and cardiometabolic complications.

This study has several strengths. First of all, the large cohort of patients enrolled, which allows to derive several interesting data; second, the importance of having collected data from a single center prospective registry and third, the excellence of the laboratory that performed the analysis of metanephrines.

Nevertheless, we should describe some study limitations. The retrospective cross-sectional design does not allow to evaluate postsurgical alterations in metabolic parameters and cardiovascular risk factors in NFAI patients. Moreover, metanephrines are not direct markers for the evaluation of sympathetic activity, because they derive from the non-neuronal metabolism of CA, which are the metabolically active hormones; in the last years more direct methods have been developed, but these techniques are not widely adopted in routine practice. It should be noted that small or non-secretive PPGL, which may bias the cardiovascular stratification, could be included in the analysis. However, the careful diagnostic work-up, the at least five years of follow-up, and the data checking through the Piedmont Oncological Network, reduced the risk of misdiagnoses. Moreover, the consistency of the obtained data suggests that these limitations were not critical for the results of the present study.

The present study showed, for the first time, a further role of a simple diagnostic tool, routinely adopted for the hormone assessment of adrenal tumors, for the cardiovascular risk stratification of patients with NFAI. Endocrine Society guidelines^3^ recommend that all patients with mildly elevated metanephrines should be followed up, because in situations of borderline positive test results and low probability of a tumor, a wait-and-retest approach can illuminate increased likelihood of an enlarging small tumor. This study went further, because our results proved that metanephrines have the capability to stratify cardiovascular risk, through their informative role about the sympathetic activity, with the intent to identify those patients who could benefit from a more aggressive or specific treatment of the known CV risk factors. Nowadays, data in literature have demonstrated that several behavioral (weight loss and sodium restriction)^44^ and therapeutic strategies (antihypertensive drugs, baroreflex activation, statins and some classes of antidiabetic agents)^45–50^ are active on sympathoinhibition. Using these strategies for patients with NFAI and high metanephrine levels, it may be possible to medically manage this additional cardiometabolic burden, though this needs to be demonstrated in interventional studies.

## Supplementary Information

Supplementary Table S1.

## Data Availability

The datasets used and/or analysed during the current study available from the corresponding author on reasonable request.
