# Supplementary material for: A retrospective study on the association between urine metanephrines and cardiometabolic risk in patients with nonfunctioning adrenal incidentaloma
Source: Sci Rep. 2022 Sep 1;12:14913. doi: 10.1038/s41598-022-19321-2 (PMC9436965; doi:10.1038/s41598-022-19321-2)
Supplement: Supplementary file 1 — Supplementary Table S1. [file 41598_2022_19321_MOESM1_ESM.docx]

| **Variables/**  **Parameters** | **Overall**  **(N=828)** | **Normetanephrine** | | | | **Metanephrine** | | | |
| --- | --- | --- | --- | --- | --- | --- | --- | --- | --- |
|  |  | **I tertile** | **II tertile** | **III tertile** | **p-value** | **I tertile** | **II tertile** | **III tertile** | **p-value** |
| Beta-blockers | 19.8% | 17.8% | 16.7% | 25.0% | 0.028†‡ | 18.3% | 21.0% | 20.0% | 0.739 |
| α-blockers | 8.6% | 5.1% | 8.0% | 12.7% | 0.005† | 7.9% | 8.8% | 9.1% | 0.875 |
| α-2 agonists | 1.4% | 1.8% | 1.8% | 0.7% | 0.467 | 0.4% | 2.6% | 1.5% | 0.095* |
| ACEi | 18.8% | 16.7% | 17.0% | 22.8% | 0.116 | 18.7% | 18.0% | 20.0% | 0.844 |
| ARB | 20.2% | 14.9% | 23.6% | 22.1% | 0.024*† | 20.5% | 21.7% | 18.5% | 0.652 |
| Thiazide (-like) diuretics | 16.1% | 14.9% | 19.6% | 13.7% | 0.133 | 22.3% | 12.5% | 13.3% | 0.002*† |
| MRA or amiloride | 5.4% | 4.7% | 4.7% | 6.9% | 0.169 | 5.0% | 2.9% | 8.3% | <0.001†‡ |
| CCB | 23.7% | 20.7% | 25.7% | 24.6% | 0.336 | 25.2% | 23.9% | 22.2% | 0.708 |
| Loop diuretics | 11.2% | 10.1% | 10.5% | 13% | 0.501 | 10.4% | 10.7% | 12.4% | 0.734 |

**Table S1.** **Classes of antihypertensive therapy according to tertiles of normetanephrine and metanephrine levels.** Abbreviations: ACEi, angiotensin converting enzyme inhibitors; ARB, angiotensin II receptor blockers; CCB, calcium channel blockers; MRA, mineralocorticoid receptor antagonist.

The table shows p-values of the statistics analyzing differences between the three tertiles.

* Significant difference between I tertile and II tertile

† Significant difference between I tertile and III tertile

‡ Significant difference between II tertile and III tertile
